# Nanotechnology-Enabled Vaccines for Malaria, Tuberculosis, HIV, and Dengue: Translational Evidence and Implementation in the Global South

**DOI:** 10.3390/nano16201326

**Published:** 2026-10-20

**Authors:** Abdelrahman Aly Mohamed, Ahmed Khairy, Mina Nageh, Ahmed Atef Mohamed, Mohamed Fawzi Hemida, Marium Aisha, Mahdi Ahmed, Lindsey Dixon, Yossef Alnasser

**Affiliations:** 1 Faculty of Medicine, Capital University, Cairo 11562, Egypt; abdelrahman.aly@med.capu.edu.eg (A.A.M.);; 2 Alexandria School of Medicine, Alexandria 21526, Egypt; 3 Faculty of Medicine, Tbilisi State University, Tbilisi 0179, Georgia; mina.nageh.mehany.hemaya207@med.tsu.ge; 4 Alexandria University, Alexandria 21526, Egypt; 5 Department of Pediatrics, BronxCare Health System, Icahn School of Medicine at Mount Sinai, New York, NY 10457, USA; 6 Faculty of Medicine, Kafrelsheikh University, Kafrelsheikh 33516, Egypt; m.ahmed38@liverpool.ac.uk; 7 American University of the Caribbean School of Medicine, Cupecoy, Sint Maarten

**Keywords:** nanovaccines, nanoparticles, tropical infectious diseases, Global South, vaccine equity

## Abstract

Nanoscale materials can support vaccine delivery, antigen display and immune stimulation, but successful platform function or enhanced immunogenicity does not necessarily establish meaningful or durable clinical protection. This narrative review examines evidence published from January 2016 through April 2026, with selected foundational studies, for nanotechnology-enabled vaccines against malaria, tuberculosis, human immunodeficiency virus (HIV) and dengue, focusing on their relevance to the Global South. These diseases were selected for their substantial public-health burden, distinct biological barriers to vaccination and contrasting stages of translational development, enabling comparison across preclinical research, human immunogenicity, clinical efficacy and implementation evidence. The synthesis distinguishes the functional contribution of nanoscale carriers, antigen-display scaffolds and adjuvants from the performance of complete vaccine formulations. Malaria has the most advanced clinical and implementation evidence among the reviewed platforms. Tuberculosis has disease-prevention evidence in a defined infected adult population, and HIV and dengue platforms remain predominantly at preclinical or early clinical stages. Clinical efficacy of a nano-enabled formulation does not, by itself, quantify the incremental benefit attributable to its nanoscale component. Across diseases, translational value depends on antigen suitability, disease-relevant immune function, durability, formulation-specific safety and applicability to the intended population. Evidence from selected trial cohorts cannot automatically be generalized across heterogeneous Global South settings. Manufacturing readiness, storage requirements, affordable complete schedules, regulatory capacity and equitable access must therefore be assessed alongside biological performance. The review identifies where links between platform function, clinical benefit and feasible implementation are supported and where they remain unestablished.

## 1. Introduction

The Global South is used here as a broad geopolitical term for diverse settings across Africa, Asia, Latin America, the Caribbean, the Middle East, and the Pacific; it is not synonymous with a single income category or epidemiological profile. Malaria, tuberculosis (TB), human immunodeficiency virus (HIV) and dengue impose a substantial burden across these settings. In 2024, the World Health Organization (WHO) estimated 282 million malaria cases and 610,000 deaths, with approximately 94% of cases and 95% of deaths in the WHO African Region [1]. WHO estimated 10.7 million incident TB cases and 1.23 million deaths, concentrated in South-East Asia, the Western Pacific and Africa [2]. The Joint United Nations Programme on HIV/AIDS (UNAIDS) estimated 40.8 million people living with HIV, including 21.1 million in eastern and southern Africa [3]. The WHO estimates there are 100–400 million dengue infections annually; a separate modeling study estimated approximately 390 million infections in 2010 [4,5]. These estimates are not additive. Surveillance, prevention and healthcare access influence both measured burden and vaccine-delivery prospects [1,2,3,4]. Across these settings, successful nanoscale delivery, antigen display or immune stimulation must be distinguished from vaccination that provides meaningful durable protection.

The four diseases present distinct biological barriers. The multistage life cycle and immune-evasion mechanisms of *Plasmodium falciparum* complicate antigen selection [6,7], whereas intracellular persistence and granuloma-associated host–pathogen interactions constrain TB vaccination [8]. HIV combines antigenic diversity, glycan shielding and restricted access to conserved envelope epitopes, with many broadly neutralizing antibody (bnAb) lineages requiring extensive maturation [9,10]. Dengue requires protection across four serotypes while accounting for antibody-dependent enhancement and baseline immune history [11,12]. Nanoscale systems offer complementary functions: lipid nanoparticles (LNPs) protect messenger RNA (mRNA) and enable antigen expression [13,14,15]; virus-like particles (VLPs) and self-assembling protein nanoparticles (SApNPs) organize repetitive antigen display [16,17,18]; polymers such as poly(lactic-co-glycolic acid) (PLGA) alter antigen delivery and release [19,20]; and liposomal or saponin-based adjuvants provide immune stimulation within clinically evaluated formulations [21,22,23,24]. These effects depend on particle composition, dimensions, geometry and administration context [16,25,26,27].

The unresolved translational question is whether the specific function of a nanoscale component supports disease-relevant immune responses, durable clinical benefit and feasible implementation. Descriptions of platform architecture and immunogenicity alone cannot establish these connections. Improved antigen expression, uptake or immune-response magnitude does not demonstrate protection, and clinical efficacy of a complete formulation does not independently quantify the contribution of its nanoscale component [13,15,18,21,22,23]. This review adds a comparative appraisal that distinguishes candidate-specific observations from mechanistic plausibility, examines evidence for incremental component effects, and separates preclinical findings, human immunogenicity, clinical efficacy and implementation evidence. The comparison is informative, because the reviewed diseases span different developmental stages: malaria has pediatric efficacy and implementation experience; TB has disease-prevention evidence in selected infected adults; HIV has early human evidence of targeted B-cell priming and maturation; and dengue platforms remain predominantly preclinical, with exploratory phase 1 findings [21,22,23,28,29,30,31].

This narrative review examines nanotechnology-enabled vaccine evidence published from January 2016 through April 2026, with selected foundational studies, for four diseases chosen for their public-health relevance, contrasting biological barriers and differing evidence maturity. It covers nanoscale carriers, antigen-display scaffolds and adjuvants; mRNA studies are included when a nanoscale delivery or antigen-display component is intrinsic to the vaccine, while other infections provide clearly identified platform context. The objective is to connect component function with disease-relevant immunity, clinical protection, durability and feasible implementation. Global South relevance is assessed within each disease through intended population characteristics, exposure setting, delivery and infrastructure requirements, product-specific manufacturing capacity, regulatory conditions, affordability and access. Recruitment region, age, host characteristics and prior infection or serostatus define direct applicability [21,22,23,28,29,30,31]. Documented outcomes are distinguished from inference and requirements not yet evaluated.

## 2. Methods

### 2.1. Review Design and Scope

This work is a critical narrative review of selected evidence on vaccines in which nanoscale components provide antigen delivery, antigen display or immune stimulation for malaria, tuberculosis, HIV or dengue. It does not seek to identify every eligible study or to estimate pooled effects. The principal scientific-literature window is 1 January 2016 to 30 April 2026; selected earlier foundational studies explain established platforms or biological principles. Epidemiological, policy, manufacturing, product and economic documents provide separately identified context. The Global South is the implementation setting of interest rather than a restriction on the geographical origin of evidence. The comparative framework distinguishes component function, demonstrated mechanisms, disease-relevant immune responses, clinical outcomes, durability, population applicability and implementation.

### 2.2. Literature Identification and Purposive Source Selection

An existing bibliography was supplemented by targeted PubMed searches and verification of individual sources. The reported PubMed revision searches were performed on 5 September 2026 using NCBI E-utilities and the scientific publication-date window specified above. One title/abstract query combined named clinical candidates with trial-related terms and retrieved 88 records; a second combined mRNA, manufacturing or technology transfer, and Global South or low- and middle-income terms and retrieved 16 records. These are query-level retrieval totals, not a count of unique screened records or included studies; overlap is not quantified. Table S4 summarizes the reported scope. Its keyword groups are descriptive and are not presented as reconstructed complete Boolean search strings. The September search date and April publication cutoff serve different purposes: the former identifies when the revision searches were performed, while the latter defines the scientific-literature window.

Sources were selected purposively for their relevance to the four diseases, the functional role of a nanoscale component, and the comparative questions of the review. Priority was given to reported human clinical outcomes, informative formulation/component comparisons, in vivo preclinical findings and candidate-specific mechanistic observations. Supporting reports were selected for population, manufacturing, economic or delivery relevance. Non-vaccine nanotherapies, duplicate reports and conference abstracts with insufficient detail were not used as disease-specific evidence. In vitro-only vaccine findings without further translational evidence were not used as disease-specific efficacy evidence. Other-disease and tumor-antigen examples provide explicitly identified platform context rather than protection evidence for the four target diseases.

**Figure 1:**
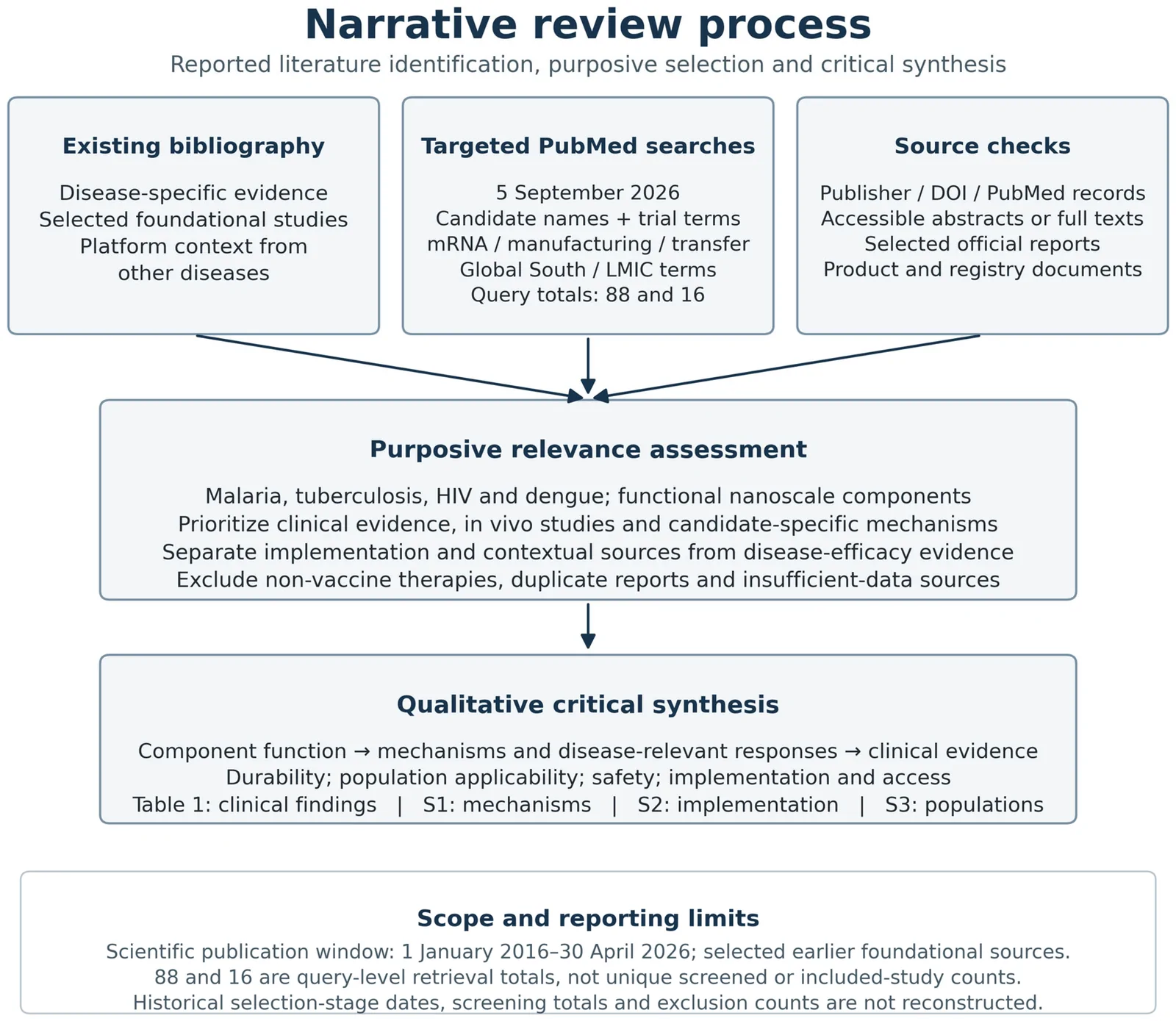
Literature identification, purposive source selection and critical narrative synthesis. The diagram shows the reported revision-search date, keyword groups, relevance criteria and analytical domains. The scientific publication window is distinct from the date of searching. Existing bibliography and contextual source checks supplement the targeted PubMed searches. Retrieval totals of 88 and 16 describe individual queries and are not unique screened-record or included-study counts. Historical selection-stage dates, exclusions and final inclusion totals are not reconstructed. The diagram describes the narrative workflow and does not assert exhaustive or PRISMA-compliant study selection.

Bibliographic identity and claim support were checked against DOI-linked publisher or Crossref records, PubMed records accessed directly or through Europe PMC, and accessible abstracts or full texts. Selected WHO, UNAIDS, Africa CDC, PAHO, Gavi–UNICEF and ClinicalTrials.gov documents were consulted for specific claims. Such checks are distinct from systematic searches of those organizations or registries; no systematic Scopus, Web of Science or registry search is claimed. Targeted implementation and product-document checks during revision supplied references [32,33,34] without extending the scientific-literature window. Source selection was purposive and did not involve dual independent screening. Figure 1 summarizes the reported narrative-review process rather than reconstructing a historical study-selection census.

### 2.3. Critical Appraisal and Narrative Synthesis

The evidence was interpreted qualitatively in relation to each study’s design, experimental model, population, comparator, endpoint and observation period. Randomized clinical efficacy findings were distinguished from early human safety/immunogenicity, animal challenge, ex vivo functional and mechanistic findings. Implementation observations were assessed separately from modeled projections, procurement announcements and policy targets. This was an endpoint-specific critical appraisal, not a formal risk-of-bias assessment or numerical quality score.

Table 1 retains trial-specific clinical endpoints, analysis populations and follow-up. Table S1 distinguishes directly observed candidate mechanisms from downstream outcomes and biological plausibility; unavailable or unresolved information is marked explicitly. Table S2 separates implementation evidence types and assumptions. Table S3 defines clinical population boundaries and extrapolation. The narrative compares which links between platform activity, clinically meaningful protection and feasible delivery are supported or unresolved. Efficacy estimates were neither pooled nor normalized onto a common scale, and neither cross-trial percentages nor platform labels were used to rank vaccines.

### 2.4. Methodological Limitations

The bibliography and targeted checks constitute a purposive narrative evidence base, not an exhaustive or reproducible study-by-study selection process. Complete historical search strategies, selection-stage dates, screening totals and exclusion counts are not reported or retrospectively reconstructed. The reference list also includes contextual and foundational documents and should not be treated as an included-study count. Candidate-focused searching can miss differently named or less advanced platforms. Although the reported revision queries applied no language filter, reliance on English-readable sources can introduce availability bias. Publication bias, selective source inclusion and underrepresentation of locally led non-English or poorly indexed research remain possible.

The review was not prospectively registered and does not claim PRISMA-compliant systematic-review reporting. There was no meta-analysis or formal risk-of-bias scoring. Uneven evidence maturity and limited representation of pregnancy and several immuno-compromised groups constrain inference. Consequently, the synthesis supports candidate-specific interpretation and identifies evidence gaps; it cannot establish complete platform coverage, comparative platform superiority or uniform applicability across Global South populations.

## 3. Defining Nanotechnology-Enabled Vaccines and Their Immune Functions

### 3.1. Operational Definition and Functional Classification of Nanotechnology-Enabled Vaccines

In this review, a nanotechnology-enabled vaccine contains a nanoscale or nanostructured component with an intended role in antigen delivery, antigen display or immune stimulation. Classification describes that component’s function within the formulation, rather than implying that nanoscale size itself confers clinical benefit. Roles are assigned separately when a vaccine combines antigen particles, carriers and adjuvants [13,17,18,19,20,24,27].

Delivery systems carry antigen or antigen-encoding cargo and influence its availability or cellular delivery, as in mRNA-loaded lipid nanoparticles and polymeric or lipid protein/peptide carriers [13,19,20,27]. Display systems organize antigens on scaffolds such as virus-like particles and self-assembling protein nanoparticles [16,17,18]. Physical display does not itself demonstrate cellular processing, major histocompatibility complex presentation or cross-presentation. Immune-stimulatory systems provide adjuvant activity, including liposomal AS01 and saponin-based Matrix-M [21,22,23,24]. Overlapping or secondary roles require candidate-specific evidence.

RTS,S/AS01 and R21/Matrix-M combine particulate antigen display with a separate adjuvant system [6,22,23]. RH5.1/Matrix-M and M72/AS01E instead combine soluble recombinant antigens with nanostructured adjuvants [21,28]. In mRNA-encoded nanoparticle vaccines, the administered lipid carrier’s delivery role differs from the encoded particle’s display role [30]. Table 1 identifies these functions; Table S1 distinguishes intended roles from measured mechanisms and outcomes.

Reported vaccine-particle dimensions may extend beyond a strict 1–100 nm range; composition, measured dimensions and function are therefore more informative than the label alone [16,27]. Neither nucleic acid nor a microneedle device is intrinsically a nanotechnology platform. Clinical efficacy establishes benefit of the tested formulation, while attribution to delivery, display, the complete adjuvant system or particle architecture requires separate evidence. The reviewed efficacy trials do not isolate nanoscale components from antigen and other constituents [21,22,23,28].

### 3.2. Mechanisms of Immunogenicity: Candidate-Specific Evidence and Limits of Inference

Table S1 distinguishes direct candidate-specific observations from downstream immunogenicity, functional assays and protection, and records composition, particle dimensions, antigen format, route and experimental model. Its selected examples do not validate a mechanism for every formulation in the same platform class.

**Antigen handling and lymphoid localization.** Dengue NS1-loaded trimethyl-chitosan particles increased intracellular antigen detection and maturation/costimulatory markers in human monocyte-derived dendritic cells ex vivo [35]. This is human-derived cellular evidence, not evidence from vaccinated humans. HIV Env protein nanoparticles showed follicular retention, follicular dendritic-cell association and particle localization in mouse lymph nodes, alongside measured germinal-center B-cell and tissue T follicular helper (Tfh) responses [18]. Follicular retention differs from conventional dendritic-cell processing; neither retention nor uptake alone establishes protection against antigen degradation.

**B-cell and Tfh endpoints.** PfCSP/Pfs25 mRNA-LNP studies measured splenic germinal-center and antigen-binding B cells [36], whereas Pvs25 studies measured memory B cells and cytokine-producing CD4-positive cells [37]. These are distinct endpoints, not interchangeable evidence of Tfh mediation. Human eOD-GT8 60mer/AS01B vaccination recruited VRC01-class precursors and supported early mutation and affinity maturation, with blood and lymph-node B-cell measurements but no detected serum neutralization [29]. Human malaria analyses used total circulating Tfh as a proxy after R21, because antigen-specific stimulation was unsuccessful, whereas RH5-specific circulating Tfh were measured after RH5.1 [38]. Neither blood readout substitutes for tissue Tfh measurement or establishes mediation of protection.

**Cellular dependence and inference limits.** CD4-positive-cell depletion abolished protection in the rodent whole blood-stage dimethyldioctadecylammonium/trehalose dibehenate (DDA/TDB) study, supporting cellular dependence of the tested formulation rather than a size-dependent liposomal mechanism [39]. Activated B-cell phenotypes described as germinal-center-like in the lipid–PLGA tuberculosis study did not demonstrate anatomical germinal centers [40]. CD8-positive responses, cytokine production and IFN-γ ELISpot results likewise do not directly establish cross presentation [35,36,41]. The Env–Gag mRNA study demonstrated VLP production in cell culture and reduced simian–human immunodeficiency virus (SHIV) acquisition in animals; it did not directly visualize particle production in vaccinated tissues or establish mucosal immune mediation [13]. Skin administration alone does not establish respiratory mucosal immunity [40]. Transmission-reduction, neutralization, animal-protection and human disease-prevention endpoints remain separate evidence levels [13,22,23,36,37,39,41].

**Figure 2:**
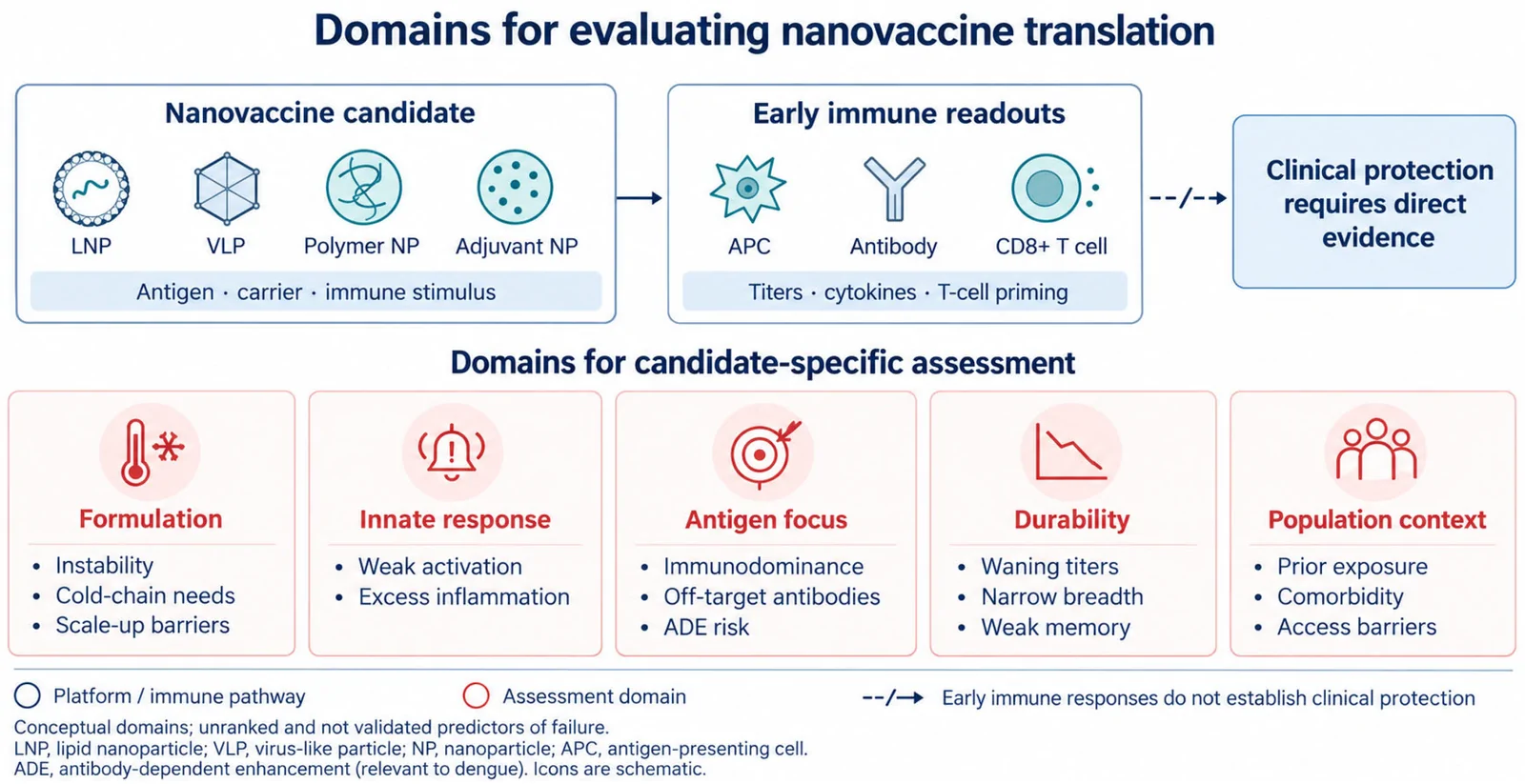
Five domains for evaluating clinical translation of nanotechnology-enabled vaccines. These conceptual domains organize candidate-level questions; they are overlapping, unranked, and not validated predictors of failure. Clinical protection requires direct evidence beyond early immune readouts. The pathways illustrate possible transitions between platform activity and protection; they are not all directly demonstrated for every reviewed candidate. Table S1 separates candidate-specific observations from mechanistic inference.

**Component attribution.** A proposed mechanism, a measured component-associated effect and clinical efficacy of a complete vaccine are different claims. Alameh and colleagues implicated an ionizable lipid component and IL-6 induction in mouse Tfh and humoral responses, supporting formulation-specific LNP adjuvant activity rather than a universal nanoscale effect or quantified human efficacy contribution [15]. Interpretation of component comparisons must retain antigen, dose, route, schedule and other adjuvant differences; changes in conformation, exposure kinetics, conjugation or tissue delivery can remain inseparable from particle incorporation. Antigen-alone, empty-carrier and soluble-antigen/scaffold controls address different attribution questions [15,42,43]. The ID93 comparison discussed in Section 3.3 tests the complete adjuvant system, while the CSP comparison in Section 4.1 tests the displayed versus mixed formulation [42,43]. Neither isolates particle architecture from all other properties. Figure 2 and Section 5 should therefore be read alongside Table S1’s candidate-specific observations and limitations.

Broader platform context includes formulation-specific LNP inflammation in mice [44], nanoparticle-mediated CD8 T-cell responses to tumor antigens [45], mucosal memory T-cell responses in experimental Chlamydia vaccination [46], and reviews of nanocarrier-based TB vaccination [47]; these sources inform platform interpretation but do not independently establish candidate-specific mechanisms or clinical protection for the vaccines reviewed here.

### 3.3. Adjuvants in Nanotechnology-Enabled Vaccine

AS01 combines monophosphoryl lipid A and QS-21 in liposomes, whereas Matrix-M is a saponin-based nanoparticle adjuvant [24]. Their clinical efficacy evidence comes from complete antigen–adjuvant formulations, as defined in Section 3.1 [21,22,23,28]. CAF01 has early human evidence of sustained antigen-specific T-cell responses rather than demonstrated disease prevention [48]. MF59 is an established oil-in-water adjuvant whose activity should not automatically be attributed to a persistent antigen depot [49].

The randomized phase 1 ID93 trial provides an antigen-alone comparator: adding GLA-SE enhanced antibody responses and the magnitude and polyfunctionality of antigen-specific CD4-positive responses [42]. Because this comparison adds both the TLR4 agonist and its emulsion, it supports an incremental effect of the complete adjuvant system, without isolating emulsion architecture or demonstrating reduced tuberculosis disease. Participant characteristics are retained in Table S3. COVID-19 mRNA-LNP efficacy similarly concerns whole products; LNP-associated immune activation is supported by separate mechanistic evidence [15,50].

The cited clinical trials report formulation-specific local and systemic reactogenicity rather than a universal tolerability ranking by adjuvant class [21,22,23,42,50]. Section 6 distinguishes platform, formulation and population safety.

### 3.4. Why Nanotechnology-Enabled Vaccines Matter in the Global South

**Thermostability and delivery.** Nanotechnology does not automatically remove cold-chain requirements. mRNA-LNP stability depends on RNA, lipids, excipients, container and formulation, while freezing can damage other freeze-sensitive vaccines [51,52,53]. Proposed stabilization approaches, including lyophilization, require measured potency and stability under the intended storage and transport conditions. Product-specific requirements are discussed in Section 7.1.

**Functional potential.** Particle composition, surface chemistry, antigen organization and adjuvant content can influence uptake and immune responses [15,16,24,26,27]; encapsulation can protect labile cargo and facilitate antigen/adjuvant co-delivery [20,27,52]. These functions provide a rationale, not proof of superior protection. Component comparisons require the candidate-specific context described in Section 3.1, Section 3.2 and Section 3.3. Route adaptation also requires joint assessment of device performance, delivered dose and storage; needle-free or intradermal administration does not itself establish cold-chain independence.

## 4. Progress in Nanotechnology-Enabled Vaccine Research (2016–2026)

**Clinical evidence and applicability. **Table 1 presents the efficacy of complete formulations: the malaria trials used rabies-vaccine controls, whereas M72/AS01E used placebo [21,22,23,28]. These comparisons do not isolate nanoscale components; informative component/formulation comparisons are appraised below using the distinctions in Section 3.

Table S3 records the recruitment region, endemicity, prior infection or serostatus, age and host characteristics. Residence in an endemic country does not establish individual prior infection, and infection-negative status does not imply no previous exposure. The results apply first to the sampled population; neither Global South recruitment nor early immune/safety findings establish effectiveness or implementation readiness [21,22,23,28,29,30,31,54,55].

Table 1 separates protective-efficacy estimates from early human immune and safety findings and retains their trial-specific interpretation limits. These include imprecision, interim follow-up, small early-phase cohorts and incompatible estimands; Section 5.3 integrates their implications across diseases [21,22,23,28,29,30,31]. This qualitative appraisal is not a formal risk-of-bias assessment.

### 4.1. Malaria

**Clinical progress.** The concentration of malaria deaths in young African children makes pediatric evidence central; the transmission intensity and seasonality affect both delivery and protection [1,56,57]. RTS,S/AS01 and R21/Matrix-M target the pre-erythrocytic circumsporozoite protein (CSP) and have demonstrated benefit in African children, including R21 efficacy at seasonal and standard sites [22,23]. Protection wanes, and additional doses help sustain it [22,23,56,58]. Trial-specific estimates, schedules and comparability limits are retained in Table 1. RH5.1/Matrix-M progressed from Tanzanian immunogenicity assessment to interim blood-stage efficacy in Burkina Faso [28,54]. These trials establish whole-formulation benefit without matched antigen-alone or alternative-adjuvant comparisons. Whole-sporozoite vaccines are an alternative antigenic approach with distinct production and administration requirements, rather than a comparator isolating nanoscale effects [59].

**Earlier platforms.** Studies have evaluated CSP display on protein nanoparticles/VLPs, VLP-coupled sporozoite peptides and transmission-blocking antigens [43,60,61,62,63], as well as LNP-delivered mRNA targeting CSP or sexual-stage antigens [36,37,64,65]. Reduced infection after challenge and transmission-reducing activity concern different outcomes: mosquito-feeding assays do not establish prevention of clinical illness. CAF01-adjuvanted whole blood-stage vaccination has also produced protective responses in rodent models [39]. These are experimental findings, not durable human efficacy evidence.

**Informative display comparisons.** Janitzek and colleagues compared VLP-displayed full-length CSP with unconjugated CSP mixed with AP205 VLPs, with equal CSP doses and aluminum hydroxide in both groups. There were increased antibody titers at several time points, including approximately 2.6-fold at seven months [43]. This supports an incremental immunogenicity effect of the tested display configuration, without isolating the particle size from conjugation/display geometry or establishing human protection. Conversely, the initial SAmut-5/3 comparison found no statistically significant liver-burden difference between soluble antigen and the tested multimeric/nanoparticle forms. The R21 benchmark’s superior result was confounded by its different antigen [60]. These complementary findings constrain claims of architecture-specific superiority.

**Population and implementation.** Transmission patterns and access to subsequent doses connect pediatric benefit to local services [22,23,56,57,58]. RTS,S/R21 require coordinated particulate-antigen/adjuvant supply, whereas RH5.1 uses a soluble protein with Matrix-M [22,23,28]. The product presentation must fit the cold-chain, staffing and visit capacity. Section 7 distinguishes RTS,S program costs from R21 supply/presentation evidence [32,33,34]. Authorization, financing, equitable schedule completion and integration with other malaria-control measures remain setting-specific [19,57,66].

### 4.2. Tuberculosis

**Clinical progress and population limits.** South-East Asia, the Western Pacific and Africa accounted for 34%, 27% and 25% of incident tuberculosis cases in 2024 [2]. Prior infection, Bacillus Calmette–Guérin (BCG) exposure and HIV status therefore matter for applicability. BCG remains the only routinely licensed TB vaccine, protecting against severe childhood disease, while pulmonary protection varies by age and setting; no validated immune correlate explains this variability universally [8,67].

M72/AS01E combines a recombinant fusion antigen with a liposomal adjuvant and demonstrated 49.7% efficacy against pulmonary TB over the three-year assessment in infected HIV-negative adults [21]. Table 1 retains the confidence intervals, analysis cohort and endpoint definition. This is disease prevention, not infection prevention or sterilizing immunity; its placebo comparison does not isolate AS01E or separate liposomes from monophosphoryl lipid A/QS-21 [21,24]. The estimate rests on 39 endpoint cases and cannot be transferred directly to uninfected people, children or people living with HIV. Phase 3 evaluation is registered as NCT06062238 [68]. A separate M72/AS01E-4 trial in South Africans aged 16–35 years with well-controlled HIV provides safety/immunogenicity evidence, not disease-prevention efficacy [55]. ID93/GLA-SE’s antigen-alone comparison and CAF01’s early human T-cell findings likewise concern immunogenicity, as discussed in Section 3.3 [42,48]. Table S3 retains the clinical population boundaries.

**Earlier platforms and component comparisons.** The Ag85B–ESAT6–Rv2034 lipid–PLGA study compared intradermal with subcutaneous administration and measured systemic antibodies and restimulated splenocyte phenotypes [40]. It did not demonstrate infection protection, inhaled delivery or respiratory mucosal immunity, and changing the route does not isolate nanoparticle incorporation. Respiratory vaccination addresses a different tissue-delivery question, requiring an active deposited dose, local tolerability and persistent tissue responses [8]; skin-delivery findings do not validate it.

Ding and colleagues compared recombinant-antigen and mi3-displayed formulations using matched antigen doses and the same AS01E-biosimilar adjuvant. The nanoparticle mixture increased cellular responses and reduced the lung bacterial burden more after challenge in mice [69]. This supports the tested display formulation within that model, without isolating size from conjugation/scaffold properties or establishing human efficacy. A separate mi3 study used an ex vivo mycobacterial growth-inhibition endpoint rather than infection-challenge or human disease prevention [70]. LNP-formulated mRNAs encoding PPE15, ESAT-6, EspC, EsxI and MetE, individually or as a mixture, also elicited responses; EsxI and the mixture reduced mouse lung burden, but boosting did not significantly improve protection over BCG [71]. Immune readouts, including interferon-gamma, remain insufficient universal surrogates [8].

**Implementation implications.** The infected HIV-negative M72 cohort does not define eligibility for every future TB vaccine [21,55]. Access to the studied two-dose schedule and follow-up must accompany assessment of prior infection, BCG exposure and HIV status. Recombinant-antigen/liposomal-adjuvant products, mi3 displays, lipid–PLGA carriers and mRNA-LNPs require distinct supply and presentation pathways [21,40,69,71]. Storage, national authorization, procurement/delivery costs and locally appropriate access remain product-specific questions; routine implementation performance for these candidates is unestablished.

### 4.3. HIV

**Developmental evidence.** The HIV burden extends beyond eastern and southern Africa to Asia, Latin America and the Caribbean [3]. The antigenic diversity, glycan shielding and conserved-envelope accessibility barriers introduced in Section 1 motivate repetitive display of engineered envelope glycoprotein (Env) immunogens and lineage-directed vaccination [9,10,18].

The Env–Gag mRNA platform demonstrated Env-bearing VLP production in cell culture and reduced heterologous SHIV acquisition in macaques [13]. Table S1 distinguishes expressed-particle dimensions from carrier dimensions and records the absence of direct particle visualization in vaccinated tissues. Other animal approaches include multilayered SApNPs [18], mRNA-encoded Env–ferritin [72], silica-coated calcium–phosphate carriers for Env DNA [73], and BG18-directed mRNA-LNP prime–boost findings in humanized immunoglobulin knock-in mice [74]. Their endpoints do not establish human protection.

**Human translation.** US IAVI G001 demonstrated VRC01-class precursor activation; G002 assessed mRNA priming and heterologous boosting in US adults, whereas G003 assessed priming in 18 HIV-uninfected adults in Rwanda and South Africa [29,30]. G003 supplies direct African priming evidence but not validation of the complete G002 boosting regimen, population-wide representativeness or protective efficacy. Trial designs and population descriptors remain in Table 1 and Table S3.

**Implementation implications.** Intended exposure patterns and individual immune repertoires must be considered alongside the feasibility of repeat heterologous doses, continuous product supply and safety follow-up. Recombinant eOD-GT8 manufacture and mRNA-LNP manufacture are distinct pathways; capacity for one does not establish capacity for the other [30,75,76]. Product-specific storage/presentation, regulation, affordability and full-regimen completion require assessment in the intended services. Durable breadth and reduced acquisition remain unproven; Section 5.4, Section 5.5, and Section 6 appraise the durability, population variation and safety.

### 4.4. Dengue Virus

**Preclinical platforms.** Dengue virus (DENV), transmitted mainly by Aedes aegypti and Aedes albopictus, is endemic in more than 100 countries, with differing serotypes, transmission patterns and immune histories across Asia, Latin America, the Caribbean, Africa and the Pacific [4]. PLGA formulations have delivered tetravalent envelope domain III (EDIII) antigens, while trimethyl–chitosan particles carried truncated DENV-2 nonstructural protein 1 (NS1) [35,77]. Molded particles and ionizable cationic LNPs delivered recombinant envelope proteins [78,79]; engineered EDIII displays and liposome-displayed envelope dimers elicited broad or type-specific mouse neutralization [80,81]. DENV-1 mRNA-LNP vaccination showed mouse protection, and modification of an enhancing DENV-2 envelope epitope improved preclinical antibody responses [82,83]. An NS1 mRNA-LNP study reported reduced viral loads and endothelial-cell cross-reactivity in experimental assays, not human safety or tetravalent protection [84]. Peptide platforms included polystyrene-linked B-/T-cell epitopes and calcium–phosphate formulations of conserved T-cell epitopes [41,85]. Polystyrene-construct neutralization differed across serotypes, and IFN-γ responses did not demonstrate cross-presentation or infection protection; Table S1 retains the construct-specific findings [41].

**Early clinical evidence.** PepGNP-Dengue, a T-cell-directed gold–peptide vaccine, was evaluated in 26 healthy dengue-naïve Swiss adults [31]. No vaccine-related serious adverse events were reported, but cellular findings were exploratory and inconsistent across dose groups. This small non-endemic cohort cannot establish protection, rare-event safety or absence of enhanced disease in previously exposed endemic populations. Trial details and population boundaries remain in Table 1 and Table S3.

**Conventional-vaccine context.** The reviewed nanoparticle candidates have no demonstrated human dengue-disease efficacy. CYD-TDV’s enhanced-disease risk in previously uninfected recipients must not be generalized to every dengue vaccine [12,86]. WHO’s 2024 position recommends TAK-003 for children aged 6–16 years in high-burden/high-transmission settings, with unresolved serotype-specific performance in baseline-seronegative recipients [86]. These observations inform population and policy interpretation; they neither establish a nanoparticle safety advantage nor authorization of investigational products.

**Implementation implications.** Eligibility and surveillance must account for age, prior serostatus, repeated exposure and circulating serotypes. A T-cell-directed candidate need not meet an antibody-focused design objective but still requires clinical protection and relevant population/serotype safety evidence [11,31,35,80,81,82,83,84]. PepGNP-Dengue used a frozen, reconstituted intradermal product [31]; research-site administration does not establish endemic-program feasibility or affordability. Storage, administration and follow-up capacity must be assessed together with product supply, national authorization, financing and access.

## 5. Cross-Disease Lessons in Nanotechnology-Enabled Vaccines

Across the four diseases, the key distinction is between successful platform activity and clinically useful protection. The discussion proceeds from platform performance and disease-relevant immune responses through clinical evidence to durability, population applicability, and implementation. These are connected questions, not a rigid sequence demonstrated for every candidate. Table S1 details candidate-specific mechanistic evidence, Table 1 clinical findings, Table S3 population characteristics, and Table S2 implementation evidence.

Figure 2 organizes five overlapping domains for candidate-level investigation. The formulation domain asks whether a stable, reproducible and biologically active product reaches the intended site. The innate-response domain asks whether activation is sufficient and appropriately regulated. The antigen-focus domain asks whether the response targets relevant protective functions. The durability domain asks whether those functions persist for the required period. The population-context domain asks whether the evidence and delivery conditions match the intended recipients. These are neither validated predictors nor mutually exclusive mechanisms or ranked causes of failure.

### 5.1. Platform Performance and Component Attribution

Successful encapsulation, antigen expression, particle uptake, repetitive display, or innate stimulation demonstrates a particular platform function; none alone establishes protection. Antigen selection and conformation, epitope accessibility, formulation stability, dose, administration route, and host context influence the meaning of that activity [15,16,18,26,27,52]. Across the reviewed diseases, clinical efficacy concerns complete antigen–adjuvant formulations, whereas selected comparator studies address narrower contributions to immunogenicity or animal protection [21,22,23,28,42,43,69]. A component-associated immune effect and a separately quantified contribution to human disease prevention are therefore distinct findings. The evidence does not identify carrier performance or antigen design as a universally dominant explanation for translational success or failure.

Comparisons that hold antigen or carrier constant or evaluate prespecified formulations and schedules can help distinguish incremental component effects. Their interpretation must still account for dose, route, schedule, follow-up, and the endpoint measured. Antigen and carrier effects therefore require joint interpretation rather than attribution of clinical benefit to particle architecture alone.

### 5.2. Disease-Relevant Immune Responses

The shared question is whether an observed response has the specificity and function required for the disease, rather than simply whether its magnitude increases. Immunodominance can direct responses toward accessible, variable, or poorly protective epitopes. For HIV, preservation of a native-like Env conformation can affect which B-cell lineages are recruited, while changes in antigen exposure may alter both desired and competing responses [10,18,74]. Relevant conformations and off-target surfaces therefore matter when interpreting Env nanoparticle responses; germline-targeting primes and subsequent boosts aim to recruit and mature specified bnAb lineages [10,18,29,30,74]. The Xie study illustrates how engineered boost immunogens can limit competing responses in humanized immunoglobulin knock-in mice, but this does not establish human protection [74]. Human precursor recruitment and early maturation likewise do not establish broad serum neutralization or protection against HIV acquisition [29,30].

For dengue, high total binding-antibody concentrations can obscure differences in neutralization across the four serotypes, functional specificity, and enhancement at antibody concentrations [11,12]. Engineered EDIII display and native-like envelope dimers offer ways to investigate antigen focus and serotype balance, but their mouse data cannot establish a safe protective response in people [80,81]. Antibody quantity should therefore be reported separately from neutralization breadth, epitope specificity, persistence, and clinical protection. T-cell-directed candidates require endpoints appropriate to their own mechanism rather than an assumed antibody threshold [31].

For tuberculosis, cellular and tissue-specific immunity, recognition of infected cells, and host–pathogen interactions remain important, and germinal-center output should not be presented as a universal bottleneck [8].

Candidate-specific cellular observations support parts of a biological framework, rather than a complete sequence demonstrated for every candidate. Table S1 distinguishes mouse lymph-node GC/Tfh observations [18], splenic GC B-cell measurements [36], human B-cell priming and lymph-node observations [29], and human malaria memory-B-cell/circulating-Tfh analyses [38]. In the latter study, the R21 Tfh readout was total circulating Tfh, whereas the RH5 readout was antigen-specific.

### 5.3. Clinical Evidence and Its Maturity

The disease areas occupy different evidentiary positions. RTS,S/AS01 and R21/Matrix-M have randomized efficacy evidence in African children, while RH5.1/Matrix-M provides interim phase 2b blood-stage efficacy evidence [22,23,28]. M72/AS01E has disease-prevention evidence in HIV-negative adults with established M. tuberculosis infection [21]. HIV studies establish human proof of concept for precursor priming and early maturation, while dengue platforms remain predominantly preclinical and PepGNP-Dengue supplies exploratory phase 1 safety and cellular-immunogenicity evidence; neither establishes human protective efficacy for the reviewed candidates [29,30,31].

These findings cannot be placed on a common efficacy scale. Clinical malaria episodes, progression to pulmonary tuberculosis, precursor activation, and cellular responses measure different outcomes, while animal challenge and ex vivo functional assays provide additional, distinct evidence. Table 1 retains trial-specific populations, endpoints, analysis sets, and observation windows. Cross-disease comparison therefore identifies which links between biological activity and clinical benefit are supported or unresolved; it does not rank platform classes from headline percentages.

The absence of additional BCG protection despite mRNA-induced immune responses and the inconsistent low- versus high-dose cellular findings in PepGNP-Dengue illustrate different unresolved links between immunogenicity and protection [31,71]. Neither observation identifies a universal cause of failure. Candidate immune measurements must be interpreted alongside prespecified clinical outcomes and mechanistic evidence without treating unvalidated markers as substitutes for protection.

### 5.4. Durability and Repeated Vaccination

For antibody-focused HIV, malaria, and dengue strategies, durable immunity can depend on antigen availability in lymphoid tissue, sustained germinal-center activity, T follicular helper support, affinity maturation, memory B-cell formation, and long-lived plasma-cell output [15,18,26,74,77]. These processes are related but distinct: a high early antibody titer does not establish persistent functional antibody, and detectable memory B cells do not by themselves establish protection during subsequent exposure. Repeated vaccination may sustain or redirect these responses, but its value must be judged against functional persistence and disease outcomes rather than a single early immune measurement [22,58].

Exploratory analyses of two phase 1b malaria trials illustrate how schedule and host context can shape these responses. For R21/Matrix-M, asparagine–alanine–asparagine–proline (NANP)-repeat-specific activated IgG-positive memory B-cell frequencies were higher in infants than adults and with 50 μg rather than 25 μg Matrix-M. For RH5.1/Matrix-M, a delayed third dose at month 6 was associated with higher antigen-specific IgG-positive memory B-cell responses than monthly or delayed fractional schedules, and these responses correlated with peak and later serum antibody measurements [38]. These small secondary analyses inform the interpretation of age, adjuvant dose, and booster timing, but their immune associations are not validated clinical correlates. They should not be conflated with the separate phase 2b RH5.1 efficacy trial, which tested a delayed third dose at month 5 [28,38].

The durability of an immune marker and the durability of clinical protection are separate evidence levels. M72/AS01E demonstrated protection over the three-year trial follow-up, without establishing its duration beyond that interval [21]. For the reviewed HIV and dengue candidates, persistent immune activity has not yet been linked to durable human protective efficacy [29,30,31]. Across diseases, repeat-dose findings must retain their specific regimen, functional endpoint, follow-up, and tolerability context before they inform expectations of durable protection.

### 5.5. Population Applicability and Public-Health Relevance

Animal and human studies differ in experimental challenge dose and route, immune repertoires, prior exposure, comorbidities, and the tissues that can be sampled. Controlled animal studies may use uniform ages and exposure histories, whereas the intended recipients may have repeated pathogen exposure, previous vaccination, chronic infection, or other conditions that modify responses [8,27,59,67]. An engineered immunoglobulin knock-in mouse can test a specified B-cell pathway but cannot establish how reliably that pathway will be recruited across naturally diverse human repertoires [30,74]. Human immunogenicity studies also differ from prevention trials conducted during natural exposure.

IAVI G001 provides a concrete human example: a subsequent analysis found that differences in VRC01-class precursor response frequencies between dose groups were better explained by IGHV1-2 genotype than by vaccine dose [87]. This supports measuring relevant immunoglobulin variation and precursor frequencies when evaluating germline-targeting vaccines. It does not justify using geography or ancestry as a biological proxy. Representative trials should characterize relevant individual exposures and immune variables and prespecify analyses of clinically meaningful heterogeneity.

Human findings apply first to the sampled population. African HIV priming evidence does not establish protection or validate the complete US boosting regimen, and findings in Swiss dengue-naïve adults do not resolve safety or efficacy in previously exposed endemic populations [30,31]. Table S3 makes these population boundaries explicit. Public-health relevance additionally depends on the local burden, exposure patterns, eligible age and infection-status groups, and access to prevention and care. Disease burden establishes the importance of a vaccine; it does not establish that a particular candidate will provide the same benefit across all intended recipients.

### 5.6. Implementation and Equitable Delivery

Affordability, cost-effectiveness and equitable access are separate questions. Affordability concerns the resources required relative to available budgets; cost-effectiveness relates incremental costs to health outcomes; equity concerns how access and benefits are distributed. Neither a favorable modeled cost-effectiveness ratio nor a low procurement price demonstrates that a country can finance delivery or reach underserved populations. We therefore distinguish documented implementation evidence, model assumptions and projections, announced procurement terms, policy targets, and proposed priorities (Table S2).

RTS,S pilot costing provides retrospective delivery-resource evidence from Malawi, Ghana, and Kenya [32]. Equivalent routine-delivery evidence is not established here for the investigational tuberculosis, HIV, or dengue candidates. Differences in evidence maturity therefore constrain comparisons of implementation performance across diseases, rather than demonstrating an intrinsic advantage of one platform class.

For R21/Matrix-M, Schmit and colleagues modeled the potential health impact and cost-effectiveness under specified epidemiological, coverage and cost assumptions [88]. These estimates are projections, not observed expenditure or access outcomes. The November 2025 Gavi–UNICEF announcement described a prospective price of US$2.99 per dose, anticipated approximately one year after the announcement [89]. It should be reported as a dated procurement agreement rather than a universally available price. Multiplying a price by the number of scheduled doses gives only a vaccine-acquisition subtotal; it excludes wastage, delivery resources and incomplete schedules. Cost per completed schedule should be distinguished from cost per case, severe outcome or disability-adjusted life-year averted. Completion does not imply complete protection.

Storage requirements, reliable supply, regulatory capacity, delivery costs, and schedule completion determine the achievable population benefit. Table S2 and Section 7 retain product- and setting-specific implementation findings and limitations. The cross-disease lesson is to relate candidate-specific clinical benefit and durability to a feasible delivery pathway while keeping observed implementation outcomes separate from projections and policy ambitions.

## 6. Safety and Population-Specific Evidence

Safety evidence is considered at three related levels: properties of the nanoscale carrier or scaffold, findings for the complete vaccine formulation, and applicability to the target population. These levels can interact and do not identify mutually exclusive causes of an adverse event. Clinical observations should therefore remain attached to the product, regimen, population, and follow-up in which they were obtained.

### 6.1. Platform-Related Safety Considerations

Platform-related considerations include the distribution, persistence, metabolism, and clearance of the carrier or scaffold. For LNPs, these properties depend on the lipid chemistry, particle characteristics, dose, and administration route [52]. PLGA is biodegradable, but that property does not establish the safety of every PLGA-containing vaccine; particle characteristics, cargo, and degradation conditions still require product-specific interpretation [20]. Protein scaffolds and non-biodegradable inorganic particles should not be assumed to share clearance behavior. Candidate-specific repeat-dose, biodistribution, and longer-term safety evidence is particularly important when multiple boosts are planned.

These material-related considerations identify questions relevant to safety assessment, rather than class-wide clinical adverse-event rates. Demonstration of tissue distribution or persistence alone does not establish clinical harm, while acceptable tolerability of one carrier-containing product does not validate every formulation using that platform.

### 6.2. Safety of the Complete Vaccine Formulation

Human clinical findings concern the administered vaccine, including its carrier or scaffold, antigen or encoded antigen, adjuvants, excipients, dose, route, and schedule. The BNT162b2 trial provides large-scale human evidence on one COVID-19 mRNA-LNP product, including common transient local and systemic symptoms [50]. Postauthorization myocarditis and anaphylaxis reports concern the COVID-19 mRNA products evaluated in those surveillance studies [90,91]. These findings do not establish the risk of every LNP formulation or isolate a causal contribution of the carrier from the RNA, antigen, other ingredients, regimen, and host factors.

Malaria and tuberculosis vaccine trials generally support acceptable tolerability for the tested regimens, with local and systemic reactogenicity relevant to the delivery program [21,22,23]. The HIV G002 skin-reaction signal warrants formulation-specific follow-up [30]. The small PepGNP-Dengue study reported no vaccine-related serious adverse events but cannot exclude uncommon events or delayed effects of the gold nanoparticle formulation [31]. Absence of a serious event in a small early-phase study is not evidence of the absence of risk. Similarly, anaphylaxis reports do not establish polyethylene glycol (PEG) as the causal component in every case [91].

### 6.3. Safety Evidence in the Target Population

Age, sex, pregnancy, immune status, prior infection or exposure, and comorbidities define important boundaries of applicability. The cited COVID-19 myocarditis surveillance identified a pattern concentrated in adolescent and young adult males; this product- and population-specific observation is not a universal property of nanovaccines [90]. A SARS-CoV-2 protein–Matrix-M study in adults with well-controlled HIV provides contextual evidence for that product and population, rather than proof that unrelated HIV or other nanovaccine candidates are safe or effective [92].

The cohorts underlying the displayed malaria efficacy estimates include African children aged 5–17 months for RTS,S, 5–36 months for R21, and 5–17 months for RH5.1 [22,23,28]. M72 efficacy concerns HIV-negative adults aged 18–50 years with established M. tuberculosis infection [21]. These descriptions help define the context of the clinical evidence; efficacy itself is not a safety endpoint, and these ages do not exhaust all safety cohorts studied within the respective programs. The separate South African M72/AS01E-4 trial supplies safety and immunogenicity evidence in virally suppressed ART-treated participants aged 16–35 years but does not establish tuberculosis disease prevention in people living with HIV [55].

### 6.4. Pharmacovigilance Across the Three Levels

Surveillance should retain the vaccine formulation, dose and schedule, target-population characteristics, event definition, and observation period. Relevant outcomes may include anaphylaxis, myocarditis for products with a corresponding signal, persistent skin reactions, and disease-specific enhanced illness [30,31,90,91]. Background event rates and the effects of infection, age, and dosing interval must be considered when interpreting postvaccination events. Temporal association or a reporting signal should be distinguished from evidence identifying a specific causal component. This approach connects platform-related questions with product-specific observations and population-specific interpretation without assigning an adverse event to an entire nanotechnology class.

## 7. Implementation Challenges Across the Global South

The implementation appraisal distinguishes documented product- and country-specific outcomes from models, procurement announcements, policy targets and requirements for future deployment. Population boundaries are detailed in Section 4, Section 5 and Section 6 and Table S3. Both geographic transfer between study and intended delivery settings and transfer between population groups within an endemic region remain relevant. The following subsections assess whether supply, regulatory arrangements, financing and delivery services can support the intended product and population; development-stage progress alone does not establish routine implementation performance.

### 7.1. Manufacturing and Infrastructure

**Manufacturing constraints depend on component function**: VLP/protein displays require consistent antigen integrity and assembly, mRNA-LNP products require coordinated cargo/carrier quality, and AS01/Matrix-M products require antigen and adjuvant supply [13,21,22,23,24,28,30,72,75]. Polymeric and peptide–inorganic constructs require their own material/antigen characterization [31,40]. These distinctions identify assessment domains, not a universal ranking of manufacturing difficulty or cost.

Readiness requires documented clinical-grade manufacture, reproducible production/release, regulatory acceptance, and sustained commercial supply and delivery. Technology transfer must identify the product, production stages, inputs, workforce, quality-management capacity and regulatory responsibilities. WHO’s mRNA program supports training and technical, quality and regulatory capacity; participation does not establish capability for every platform or commercial availability of a reviewed candidate [76]. Partnerships and investment scenarios should be assessed against a sustained quality-assured supply [76,93].

**Presentation determines infrastructure needs.** eOD-GT8 clinical material was stored at −80 °C, without establishing its suitability for routine refrigerated distribution [75]. The WHO-listed single-dose R21 product is a ready-to-use liquid stored at 2–8 °C, with a 24-month shelf life and secondary-packaging volume of 14.06 cm^3^/dose [33]. The two-dose instructions require opened vials to be discarded within six hours or at session end, whichever occurs first [34]. These are presentation-specific requirements, not measured program wastage.

The manufacturing cost differs from the procurement price. The cited manufacture/investment reports do not establish audited commercial cost-of-goods estimates for every candidate at a named Global South production site; so, a local-manufacturing cost advantage cannot be quantified [75,93].

### 7.2. Financing, Procurement, and Delivery Costs

Table S2 presents country-specific RTS,S pilot estimates for delivery resources and four-dose completion in Malawi, Ghana and Kenya [32]. Their applicability depends on product, costing perspective/year and program conditions; they should not be transferred unchanged to R21 or investigational TB, HIV or dengue products.

Financial expenditure differs from economic resource use: donated vaccine can carry substantial resource value despite no recipient acquisition expenditure [32]. Budget-impact assessments must specify the procurement terms, donor/domestic contributions, recurrent and infrastructure costs, eligible recipients and completion, including whether household travel and caregiver time are excluded. Favorable cost-effectiveness does not establish budget affordability.

The R21 model, procurement announcement and pilot costing answer different questions [32,88,89]. One should retain model assumptions/sensitivity ranges, agreement dates/conditions and delivery-cost boundaries rather than combining them into an apparently observed cost of protection. Section 5.6 explains the economic distinctions; Table S2 preserves the detailed evidence and assumptions.

### 7.3. Regulatory and Governance Pathways

Regulatory milestones differ from review durations. R21’s WHO prequalification date, 19 December 2023, does not measure national submission-to-approval or the rollout time [33]. The East African Community medicines-harmonization overview reports approximately halved average registration time during its pilot, but this is not a measured review-time reduction for the four reviewed disease-specific vaccine groups [94]. WHO Global Benchmarking Tool maturity levels characterize regulatory systems, not individual candidate approval or speed [95].

Comparable country-specific regulatory durations for these investigational products remain unestablished. Monitoring should therefore distinguish submission, national authorization, prequalification where applicable, procurement and first program use, identifying the product and country rather than inferring timelines from regional capacity measures.

### 7.4. Efficacy, Durability, and Multidose Schedules

Durability affects visit frequency and the resources needed for a completed schedule. The WHO recommends four malaria doses beginning at around five months, with a fifth considered in selected high-risk settings [57]. M72/AS01E efficacy was tested with two doses one month apart, while experimental HIV regimens may require sequential heterologous boosts [21,30]. Additional visits create staff and household demands and opportunities for missed doses. Programs should report initiation, completion, time spent incompletely vaccinated and the cost per completed schedule alongside cases or severe outcomes averted. Completion does not imply complete individual protection. Higher durability, feasible coadministration, reliable dose tracking and schedules compatible with local services are implementation priorities; the underlying efficacy and population limits remain those described in Section 4, Section 5 and Section 6.

### 7.5. Cross-Regional Disparities in Implementation Progress

African RTS,S programs document delivery resources/costs, whereas India’s R21 product record documents presentation and supply requirements—different implementation milestones [32,33,34]. Variation among Malawi, Ghana and Kenya in staffing, cold-chain requirements and costs limits continent-wide generalization (Table S2). Routine-delivery evidence for investigational TB, HIV and dengue candidates is lacking; so, maturity also confounds geographical comparisons.

Capacity initiatives address different constraints: Africa’s PAVM framework sets a 60% local-manufacturing target for 2040 from a historical baseline below 1%, Korea’s WHO hub addresses workforce training, and PAHO’s Regional Revolving Funds support procurement cooperation [96,97,98]. Technology-transfer participation and investment scenarios concern capacity development not achieved candidate-specific supply or access [76,93].

The sources do not support an overall regional ranking. Comparisons require matched product, population/exposure, burden, schedule, costing perspective and observation period; sustained supply, completed-schedule costs, national regulatory durations and equitable uptake remain incompletely documented [32,94,95]. Within-country initiation and completion by geography and service access are needed to assess equitable delivery.

## 8. Future Directions

The priorities below follow from incomplete component attribution, unvalidated immune surrogates, limited durability and population evidence, and gaps in manufacturing and delivery data. Their relevance depends on candidate maturity: mechanistic findings, early human responses, clinical protection and routine implementation answer different questions.

### 8.1. Antigen–Nanoplatform Relationships

Future evidence should identify whether the nanoscale component delivers cargo, displays antigen or supplies adjuvant activity and interpret that role alongside antigen suitability, conformation and epitope accessibility. Reports of component-associated effects should state the comparator, endpoint and formulation differences that remain inseparable [42,43,69]. Efficacy of a complete product is clinically useful even when its nano-specific contribution has not been separately quantified; that contribution nevertheless cannot be inferred from efficacy alone [21,22,23,28].

mRNA-LNP approaches support the development of sequential HIV immunogens and dengue antigens, and some constructs encode self-assembling antigen displays [13,30,72,74,82,83,84]. The administered carrier must be distinguished from the encoded display. Mixing immunogens in one dose should not be assumed to reproduce the biology of sequential heterologous boosting.

### 8.2. Functional Immune Evidence and Durability

Priorities should follow the disease and candidate mechanism. For malaria, clinical protection and its persistence across exposure settings must remain distinct from blood-stage functional assays and transmission-blocking endpoints [22,23,28,56]. For TB, cellular responses and animal or ex vivo outcomes require interpretation against prevention of human disease [21,42,69,70]. For HIV, precursor recruitment and early maturation must be connected to durable functional breadth and ultimately reduced acquisition [29,30]. For dengue, relevant evidence includes serotype-specific neutralization and enhancement for antibody-directed candidates, cellular function for T-cell-directed candidates, and NS1-associated cross-reactivity where applicable, alongside clinical protection and safety [11,31,35,80,81,82,83,84].

Follow-up should distinguish the persistence of an immune marker from the persistence of clinical benefit and report the uncertainty in terms of the duration of protection and the need for additional doses. Functional responses, repeat-dose tolerability and schedule completion should inform the interpretation of durability; a single early immune measurement cannot establish sustained protection.

### 8.3. Representative Populations and Intended Services

Evaluation priorities should reflect pediatric age and transmission patterns for malaria; infection, BCG exposure and HIV status for TB; exposure and immune-repertoire diversity for HIV; and serostatus, age and circulating serotypes for dengue [21,22,23,30,31,55,57,86,87]. Pregnancy and other underrepresented host conditions remain product-specific evidence gaps. Local research participation and leadership should help define relevant population and service questions, without treating enrolment in one country as representation of an entire region. Assessment should connect clinical eligibility to access to visits, follow-up and completion, including underserved groups.

### 8.4. Manufacturing, Delivery, and Equitable Implementation

Manufacturing assessments should follow the platform-function distinctions in Section 7.1 and document production, release and sustained-supply milestones. Product presentation and stability must be related to intended storage and delivery services; transfer announcements and investment scenarios cannot substitute for product-specific evidence [33,34,75,76,93]. Regulatory assessments should distinguish observed milestone dates from forecast timelines.

Implementation reporting should include the actual procurement expenditure, delivery and household costs, temperature excursions, wastage, staff workload and dose completion, along with the uncertainty in projected health benefits. Economic evaluations should distinguish financial from economic costs and specify perspective, currency year, time horizon, comparator and financing assumptions. Equity assessment should identify who initiates and completes vaccination and who remains underserved. Local manufacture or a lower price does not establish access. These priorities complement candidate-specific efficacy, safety and durability evidence; where country/product data are unavailable, implementation performance remains unestablished.

## 9. Conclusions

Nanotechnology can protect an antigen, organize particulate display and provide immune stimulation. Its translational value, however, cannot be established by nanoscale presence or improved immunogenicity alone. This review’s principal contribution is to distinguish between platform function, disease-relevant immune responses, clinical benefit, durability, population applicability and implementation and to identify where the evidence connects these domains. The efficacy of a complete formulation remains distinct from attribution of its benefit to its nanoscale component.

Malaria’s clinical and implementation evidence, TB disease-prevention evidence in infected HIV-negative adults, and the earlier HIV and dengue findings support a candidate-specific interpretation rather than a ranking of nanotechnology classes. Meaningful translation requires connecting the component’s particular role within the vaccine to durable clinical outcomes and realistic delivery in the intended population. Antigen and formulation suitability, representative evaluation, reliable manufacture, affordable complete schedules, regulatory and public-health capacity, local research leadership and measured access outcomes determine the relevance across the heterogeneous Global South.

## Figures and Tables

**Table 1:** Selected clinical evidence for nano-enabled vaccines, with trial-specific endpoints and limits to quantitative comparability. (**A**) presents clinical efficacy estimates; (**B**) presents early safety and immunogenicity findings. Estimates retain the populations, endpoints and observation periods of the cited reports; they have not been mathematically normalized, pooled or ranked.

**(A) Clinical Efficacy Evidence**							
**Candidate/Nanoscale Component and Functional Role**	**Population, Infection Status and Exposure Setting**	**Schedule and Comparator**	**Analysis Population**	**Exact Endpoint and Effect Measure**	**Time Origin and Follow-Up**	**Reported Efficacy and Analysis Status**	**Quantitative Comparability and Interpretation Limits**
RTS,S/AS01; malaria. Antigen display: particulate CSP antigen. Immune stimulation: liposomal AS01 [22]	Children aged 5–17 months; 8922 across three arms; 11 sites in seven African countries with heterogeneous malaria transmission. Endemic-exposure cohort, not selected for malaria-naïve serostatus.	Months 0, 1, 2 and 20 (R3R); versus non-malaria comparator regimen (C3C; rabies vaccine in this age cohort).	Modified ITT: participants receiving ≥ 1 dose; estimate compares R3R with C3C.	All clinical malaria episodes, including recurrences: illness with axillary temperature ≥ 37.5 °C and *P. falciparum* asexual parasitemia > 5000/µL or protocol-defined severe malaria; passive detection. VE from negative binomial regression of episode rates.	Dose 1 (month 0) to study end; median 48 months. Includes primary-series and post-booster periods.	36.3% (95% CI 31.8–40.5). Final phase 3 analysis.	Not directly comparable with R21/RH5.1: recurrent-event rate rather than first-event hazard, modified ITT rather than per-protocol analysis, earlier time origin and longer observation. Age, transmission, schedule and case definitions also differ.
R21/Matrix-M; malaria. Antigen display: particulate CSP antigen. Immune stimulation: saponin-based Matrix-M [23]	Children aged 5–36 months; Burkina Faso, Mali, Kenya and Tanzania; two seasonal and three perennial (“standard”) sites. Endemic-exposure cohort, not selected for malaria-naïve serostatus.	Three doses four weeks apart; booster 12 months after dose 3. Licensed rabies vaccine (Abhayrab) control.	Modified per-protocol, *n* = 4644 (3103 vaccine; 1541 control): three correct doses within specified intervals.	Time to first clinical malaria episode: axillary temperature ≥ 37.5 °C or fever history within 24 h, plus *P. falciparum* asexual parasitemia > 5000/µL; passive detection. VE = 1−Cox hazard ratio; seasonal and standard sites analyzed separately.	Day 14 after dose 3 to 12 months after dose 3; before booster. Report also includes 18-month seasonal-site follow-up.	75% (95% CI 71–79), seasonal; 68% (61–74), standard. Completed 12-month primary analysis; longer trial follow-up ongoing in the cited report.	Seasonal/standard estimates share definitions and analysis but represent different exposure and delivery settings, not randomized setting comparisons. Not directly comparable with RTS,S/RH5.1 because age, schedule, exposure, analysis and follow-up differ; cannot establish superiority.
RH5.1/Matrix-M; malaria. Immune stimulation: saponin-based Matrix-M; recombinant RH5.1 is a soluble antigen, not a nanoparticle [28]	Children aged 5–17 months; 361 enrolled across two cohorts in Nanoro, Burkina Faso; seasonal transmission. Endemic-exposure cohort, not selected for malaria-naïve serostatus.	Delayed: months 0, 1, 5; monthly: months 0, 1, 2. Rabivax-S rabies vaccine on corresponding schedules; each vaccine regimen compared with pooled control cohorts.	Per-protocol, *n* = 338: 114 in the delayed RH5.1/Matrix-M group, 112 in the monthly RH5.1/Matrix-M group, and 112 pooled rabies controls (55 delayed; 57 monthly). Participants received three vaccinations correctly within the prespecified intervals. Each vaccine regimen was compared with the pooled controls [28].	Time to first clinical malaria episode: axillary temperature ≥ 37.5 °C and/or fever history within 24 h, plus *P. falciparum* asexual parasitemia > 5000/µL; passive detection. VE = 1 − Cox hazard ratio.	Day 14 to six months after dose 3; vaccination completed partway through the malaria season.	55% (95% CI 20–75), delayed; 40% (−3 to 65), monthly. Interim phase 2b analysis; follow-up ongoing.	Not directly comparable with R21 despite a similar first-event definition: shorter follow-up, one seasonal setting, different schedules and pooled controls. Regimens were evaluated in separate cohorts; headline estimates do not establish delayed-regimen superiority. Monthly CI includes no effect.
M72/AS01E; tuberculosis. Immune stimulation: liposomal AS01E; M72 is a recombinant fusion antigen [21]	HIV-negative adults aged 18–50 years; IGRA-positive *M. tuberculosis* infection, without active TB; baseline sputum PCR-negative. Kenya, South Africa and Zambia; TB-endemic settings.	Two intramuscular doses one month apart; placebo control.	According-to-protocol efficacy cohort, *n* = 3289 (1626 vaccine; 1663 placebo).	First bacteriologically confirmed pulmonary TB episode not associated with HIV infection: clinical suspicion plus positive sputum culture and/or PCR, with sputum obtained before TB treatment (first case definition). VE = 1 − Cox hazard ratio.	Day 30 after dose 2 to the three-year assessment (month 36 after dose 2).	49.7% (primary 90% CI 12.1–71.2; 95% CI 2.1–74.2 also reported); 13 versus 26 cases. Final three-year phase 2b analysis.	Not quantitatively comparable with malaria estimates: different pathogen, adult population, pre-existing infection, disease endpoint and observation period. Measures prevention of TB disease in infected adults, not acquisition of infection. Only 39 endpoint cases; wide CI and limited generalizability to uninfected people, children or people living with HIV.
**(B) Early Clinical Evidence Without Protective-Efficacy Estimates**							
**Candidate/Nano Component**		**Trial Population and Schedule**	**Comparator/Study Design**	**Reported Immune or Safety Finding**		**Quantitative Comparability and Interpretation Limits**	
eOD-GT8 60mer/AS01B; HIV; IAVI G001. Antigen display: 60mer protein nanoparticle. Immune stimulation: liposomal AS01B [29]		48 healthy adults; 36 received vaccine. Two priming doses eight weeks apart.	Randomized, double-blind, placebo-controlled phase 1 trial.	VRC01-class bnAb precursors induced in 97% of vaccine recipients.		Immune-response proportion, not VE or protection against HIV acquisition; no clinical efficacy endpoint or efficacy follow-up window. Cannot be compared quantitatively with Panel A.	
mRNA-encoded nanoparticles; HIV; IAVI G002/G003. Delivery: LNP carrier of mRNA. Antigen display: encoded protein nanoparticles [30]		G002: 60 US adults in priming/boosting groups. G003: 18 adults in Rwanda and South Africa; two priming doses eight weeks apart.	G002: randomized, open-label regimen groups. G003: single-arm, open-label.	G002: precursor induction and early maturation after heterologous boosting. G003: priming evidence. Skin reactions in 18% of G002 participants.		Immunogenicity and adverse-event findings, not HIV-acquisition efficacy. No protective-efficacy estimate, clinical efficacy endpoint or efficacy follow-up window; incomparable with Panel A. Further maturation and safety evaluation needed.	
PepGNP-Dengue; dengue. Peptide carriage/display: gold nanoparticles bearing peptide antigens; a T-cell-directed formulation, without independently demonstrated gold-specific immune stimulation [31]		26 dengue-naïve Swiss adults aged 18–45 years; 20 received vaccine. Two intradermal doses 21 days apart.	Double-blind, randomized, vehicle-GNP-controlled phase 1 trial.	Exploratory low-dose cellular response; no vaccine-related serious adverse events reported. Immune assessments through day 180 after dose 1.		Non-endemic, dengue-naïve cohort; small safety/immunogenicity trial with inconsistent dose response. No clinical dengue-efficacy endpoint or protective-efficacy estimate; incomparable with Panel A.	

Table notes. Analysis populations and time windows apply specifically to the estimates displayed, not to every analysis in each trial. “Final” and “ongoing” describe the status at publication of the cited report. For malaria, endemic exposure does not imply a standardized baseline infection or serostatus across trials. Similar fever and parasitemia thresholds do not harmonize recurrent-event and first-event estimands, surveillance periods, transmission, background preventive interventions or analysis populations. These aggregate results therefore do not support a common normalized efficacy scale, pooled estimate or cross-vaccine ranking. For M72/AS01E, the primary inferential interval is the 90% CI; the published 95% CI is also retained for transparency. Confidence-interval widths should be interpreted considering their nominal confidence levels. Panel B percentages describe immune responders or adverse events, not vaccine efficacy. Clinical efficacy pertains to the complete formulation and does not quantify the incremental contribution of its nanoscale component [21,22,23,28]. Abbreviations: AS01, adjuvant system 01; bnAb, broadly neutralizing antibody; CI, confidence interval; CSP, circumsporozoite protein; eOD-GT8, engineered outer domain germline-targeting version 8; HIV, human immunodeficiency virus; IAVI, International AIDS Vaccine Initiative; IGRA, interferon-gamma release assay; ITT, intention-to-treat; LNP, lipid nanoparticle; mRNA, messenger RNA; PCR, polymerase chain reaction; RH5, reticulocyte-binding protein homologue 5; TB, tuberculosis; VE, vaccine efficacy.

## Data Availability

No new data were created or analyzed in this study. Data sharing is not applicable to this article.

## Supplementary Materials

The following supporting information can be downloaded at https://www.mdpi.com/article/10.3390/nano16201326/s1, Table S1: Candidate-specific mechanistic evidence for selected reviewed vaccine platforms; Table S2: Implementation evidence and limits of inference; Table S3: Geographic, exposure and host characteristics of the disease-specific clinical evidence discussed; Table S4: Descriptive scope of literature identification, purposive source selection, and critical narrative synthesis.
