# Supplementary material for: Nanotechnology-Enabled Vaccines for Malaria, Tuberculosis, HIV, and Dengue: Translational Evidence and Implementation in the Global South
Source: Nanomaterials (Basel). 2026 Oct 20;16(20):1326. doi: 10.3390/nano16201326 (PMC13708461; doi:10.3390/nano16201326)
Supplement: Supplementary file 1 [file nanomaterials-16-01326-s001.zip › nanomaterials-4603206-supplementary.pdf]

Supplementary Tables

Table S1. Candidate-specific mechanistic evidence for selected reviewed vaccine platforms

Representative candidates were selected to cover the four target diseases, complementary delivery or cellular observations, and informative formulation comparisons. Clinical formulations are included to distinguish clinical efficacy from mechanistic validation. Size values describe the specified antigen particle, carrier, or adjuvant; these objects are not interchangeable. DLS and electron-microscopy dimensions are method-dependent and are not directly interchangeable. A reported platform dimension does not necessarily represent measurement of the administered clinical lot.

Evidence terms: Direct observation identifies a measured cellular or tissue endpoint, not necessarily a causal mechanism. **Dependence** identifies an intervention supporting the requirement for a cell population in the tested model. **Immunogenicity/function** and **protection** are downstream endpoints. **NR-S** means not reported in the source set examined for that row; it does not mean absent from all publications. "Not demonstrated here" is restricted to those sources. Values following ± are reproduced as reported, without assuming that every paper uses the same dispersion measure.

A. Malaria

Candidate / original references	Composition and reported size	Antigen format	Route	Direct cellular or mechanistic evidence	Immune or protection endpoint	Model and interpretation limit
RTS,S/AS01 [22]	HBsAg-based antigen particles with liposomal AS01; numerical particle dimensions NR-S	CSP–HBsAg fusion coassembled with HBsAg	IM	Clinical efficacy trial does not directly test uptake, lymph-node trafficking, GC/Tfh mediation or cross-presentation	Anti-CSP responses and clinical-malaria efficacy	Humans; phase 3 trial. Evidence concerns the complete formulation, not an isolated nanoparticle contribution
R21/Matrix-M [23, 38]	CSP–HBsAg VLP plus saponin-based Matrix-M. Trial appendix describes R21 as **~23 nm**; this is a reported platform dimension, not a	Particulate recombinant CSP fusion	IM in phase 3 [23]; cellular report [38] analyzes a separate phase 1b cohort	**Direct observation:** antigen-specific memory B cells; **total**, not CSP-specific, blood cTfh	Anti-NANP responses; clinical-malaria efficacy [23]. Cellular study reports	Humans; clinical and cellular findings come from different trials. Neither establishes

	clinical-lot measurement. Matrix-M dimensions NR-S			used as a proxy [38]	age/adjuvant-dose-associated B-cell differences [38]	causal mediation by cTfh or particle size
RH5.1/Matrix-M [38, 54]	Soluble RH5.1 protein with saponin-based Matrix-M; adjuvant dimensions NR-S. RH5.1 itself is not a nanoparticle	Recombinant soluble RH5.1	IM [54]	**Direct observation:** RH5-specific memory B cells and antigen-specific cTfh responses; regimen/exposure-associated differences [38]	Antibody responses; RH5-specific memory B cells associated with peak and later serum antibody levels [38]	Humans; phase 1b cellular analyses. Blood responses are not direct GC-Tfh measurements or proof of a nano-specific causal effect
CSP–AP205 VLP [43]	AP205 scaffold with covalently attached CSP; TEM **~49 nm** decorated particles versus **~30 nm** unconjugated Spy-VLP; alum-adjuvanted	Recombinant CSP ectodomain, termed “full-length CSP” by authors, coupled to VLP	IM	Conjugated versus soluble CSP mixed with VLPs supports display-associated immunogenicity. Uptake, GC/Tfh and trafficking not directly demonstrated here	Higher anti-CSP IgG at several time points; persistent responses through day 212; subclass profiling	BALB/c mice. Same-alum comparator does not isolate particle size. No infection-protection endpoint in this study
PfCSP/Pfs25 mRNA-LNP [36]	Ionizable lipid, phosphatidylcholine, cholesterol and PEG-lipid carrier; DLS mean hydrodynamic diameter **~80 nm**	Encoded PfCSP or Pfs25, assessed separately and together	IM	**Direct observation:** splenic GC B cells and antigen-binding B cells; Pfs25-specific cytokine-producing	Antigen-specific antibodies; Pfs25 transmission-reducing activity; PfCSP	BALB/c mice; cellular analyses also C57BL/6. GC and antigen-binding B

				CD4/CD8 cells. GC gating reported in the supplementary material	protection in mouse infection experiments	cells were separately measured. Do not infer Tfh mediation or cross-presentation; some later protection assessments followed earlier challenge exposure
Pvs25 mRNA-LNP [37]	Ionizable lipid, phosphatidylcholine, cholesterol and PEG-lipid carrier; size **not reliably extractable** because publication prints "~80 nM" as a diameter†	Encoded Pvs25; recombinant protein/ISA-51 and mixed-regimen comparators	IM	**Direct observation:** antigen-specific splenic memory B-cell ELISpot and cytokine-producing CD4 cells; GC/Tfh or trafficking not directly demonstrated here	Antigen-specific IgG, native-antigen recognition and durable transmission-reducing activity through seven months after boosting	BALB/c mice; functional assays use patient-derived infected blood, not vaccinated humans. Comparator differs in antigen format and adjuvant as well as delivery platform
Whole blood-stage vaccine with DDA/TDB or CAF01 [39]	Cationic liposomes containing DDA and TDB; in-house DDA/TDB and supplier CAF01 evaluated.	Killed whole asexual blood-stage parasite material	SC for mechanistic experiments; separate IM comparison	**Dependence:** CD4-cell depletion abolished protection; B-cell-deficient mice were also unprotected. These interventions	Control of parasitemia, survival and cross-strain protection	Rodent malaria models, including BALB/c and C57BL/6-derived models. Key depletion experiments

	Dimensions NR-S			support cellular requirements within the tested model		used in-house DDA/TDB; do not assume all experiments used supplier CAF01 or that liposome size caused protection

B. Tuberculosis

**Candidate / original references**	**Composition and reported size**	**Antigen format**	**Route**	**Direct cellular or mechanistic evidence**	**Immune or protection endpoint**	**Model and interpretation limit**
ID93/GLA-SE [42]	TLR4 agonist GLA in stable oil-in-water nano-emulsion; numerical droplet dimensions NR-S	Recombinant ID93 fusion protein	IM	**Direct observation/comparator:** increased polyfunctional CD4 responses and antibody Fc-mediated effector activity with GLA-SE versus ID93 alone	Higher antibody titers, altered subclasses and functional antibody/cellular responses	Humans; phase 1. Supports contribution of the complete adjuvant system; does not isolate emulsion structure from GLA activity. No disease-efficacy endpoint
Lipid–PLGA hybrid vaccine [40]	PLGA core/lipid shell (DOPC, DOPE, DOBAQ, EPC), with	Ag85B–ESAT6–Rv2034 fusion protein	ID versus SC	**Direct observation:** route-associated polyfunctional CD4/CD8 and activated B-cell phenotypes in ex vivo restimulated	Antigen-specific serum IgG and cellular responses	C57BL/6 mice. Matched-route comparison does not isolate a

	CpG/MPLA. DLS Z-average: **143.9 ± 18.5 nm (SC)**; **143.1 ± 18.5 nm (matched-antigen ID)**; **156.4 ± 14.7 nm (lower-antigen ID)**			splenocytes. "GC-like" interpretation is not direct evidence of anatomical GCs		nanoparticle effect. No infection challenge or respiratory mucosal immunity demonstrated

C. HIV

**Candidate / original references**	**Composition and reported size**	**Antigen format**	**Route**	**Direct cellular or mechanistic evidence**	**Immune or protection endpoint**	**Model and interpretation limit**
Env–Gag VLP mRNA [13]	LNP carrier containing ionizable lipid, phospholipid, cholesterol and PEG-lipid; numerical LNP diameter NR-S. Expressed VLPs: EM mean **~90 nm**, range **65–110 nm**, in cell-culture preparations	mRNA encoding HIV Env and SIV Gag	IM	**Direct observation:** VLP production/release in cell culture; Env-specific blood cTfh and T-cell responses in macaques. In vivo VLP formation was not directly visualized	Env antibodies, heterologous tier-2 neutralization and reduced/delayed SHIV acquisition	Cell culture, BALB/c mice and rhesus macaques. Some macaque regimens included protein boosts. Tfh–protection association required outlier exclusion; no established mucosal immune mechanism

Env protein SApNPs [18]	Env-displaying E2p and I3-01v9 protein scaffolds; DLS **51.9–69.0 nm for E2p** and **51.1 nm for I3-01v9**. Ferritin comparator: **41.9 nm**. Values characterize specified constructs	Multivalent recombinant Env trimers	Footpad injection for mouse lymph-node mechanism studies; separate NHP regimens used SC or IM	**Direct observation:** follicular retention, FDC association and particle localization by imaging/TEM; lymph-node GC B-cell and Tfh responses by histology/flow cytometry	Lymphoid-tissue responses in mice; separate serological/neutralization assessments in mice, rabbits and NHPs	Mechanistic tissue evidence is **mouse** evidence. Do not transfer it to NHPs or humans. Construct/adjuvant differences prevent attribution to size alone
eOD-GT8 60mer/AS01B [29]	Lumazine-synthase-based 60mer antigen particle plus liposomal AS01B; numerical dimensions NR-S	Multivalent germline-targeting outer-domain immunogen	IM	**Direct observation:** intended VRC01-class precursor recruitment; vaccine-specific lymph-node GC B cells and blood B cells; early somatic mutation/affinity maturation	VRC01-class responses in 35/36 vaccine recipients; **no detected serum neutralization** against tested viruses	Humans; IAVI G001 phase 1. Demonstrates germline-targeting priming, not broadly protective immunity or a size-dependent delivery mechanism

D. Dengue

Candidate / original references	Composition and reported size	Antigen format	Route	Direct cellular or mechanistic evidence	Immune or protection endpoint	Model and interpretation limit

Truncated NS1–TMC [35]	Trimethyl-chitosan particles; reported mean **282.7 ± 1.65 nm** by particle-size analysis	Encapsulated recombinant truncated DENV-2 NS1	**Intraperitoneal** in mice; no vaccination route for human ex vivo cells	**Direct observation:** intracellular NS1 in human-derived MoDCs; CD83, CD80, CD86 and HLA-DR responses; cytokine/chemokine secretion. Mouse IFN-γ-positive CD8 responses also measured	Anti-NS1 IgG/subclasses; serum-mediated complement-dependent effects on infected cells; mouse cellular responses	Human **ex vivo** MoDCs plus BALB/c mice. No human vaccination efficacy; CD8 response alone does not establish cross-presentation
Peptide–polystyrene particles [41]	Nominal bare particle **50 nm**; conjugate DLS: **98.1 ± 1.9 (NP1), 100.8 ± 2.9 (NP2), 63.1 ± 1.1 (NP3), 63.4 ± 1.4 nm (NP4)**	Linked synthetic B-cell, CD8-epitope and helper-epitope peptides coupled to particles	SC	Antibody and splenocyte IFN-γ ELISpot responses support immunogenicity; responding T-cell subsets and cross-presentation were not established by ELISpot	**NP3/NP4** sera neutralized all four serotypes; NP1/NP2 lacked detectable DENV-1/2 neutralization	BALB/c mice and serum/cellular assays. No infection-challenge protection or ADE evaluation demonstrated

Table footnotes

- † The reviewed publication [37] reports the unit "nM". This is dimensionally incompatible with a diameter; the table therefore does not silently substitute "nm" or borrow [36]'s value. The qualitative findings in [37] do not depend on resolving this typographical issue.
- NR-S is a reporting limitation of the checked sources, not a statement that a measurement was never made. In particular, [13] states that LNP size was tested but provides no numerical carrier diameter in the reviewed main text and supplement.
- The splenic GC phenotype in [36], lymph-node GC/Tfh observations in [18, 29], memory B-cell assays in [37, 38], and circulating Tfh in [13, 38] are distinct endpoints. The table does not equate them.
- A serum neutralization assay, transmission-reduction assay, ex vivo infected-cell assay, animal infection endpoint and human clinical endpoint are not interchangeable evidence levels.

- No selected study's downstream CD8 or IFN-γ response is treated as sufficient proof of cross-presentation. No route of administration alone is treated as proof of mucosal immunity.

Abbreviations: AS01, liposomal adjuvant system; CAF01, cationic adjuvant formulation; CSP, circumsporozoite protein; cTfh, circulating T follicular helper cells; DDA, dimethyldioctadecylammonium; DLS, dynamic light scattering; EM/TEM, electron/transmission electron microscopy; FDC, follicular dendritic cell; GC, germinal center; HBsAg, hepatitis B surface antigen; ID/IM/SC, intradermal/intramuscular/subcutaneous; LNP, lipid nanoparticle; MoDC, monocyte-derived dendritic cell; NHP, nonhuman primate; PLGA, poly(lactic-co-glycolic acid); SApNP, self-assembling protein nanoparticle; TDB, trehalose dibehenate; TMC, trimethyl chitosan; VLP, virus-like particle; ADE, antibody-dependent enhancement.

Table S2. Implementation evidence and limits of inference

A. Country-specific RTS,S pilot estimates

Setting: Malawi, Ghana and Kenya pilot implementation, 2019–September 2021; government perspective; **2020 US dollars**. Estimates combine retrospective resource/expenditure data with valuation and allocation assumptions [32].

Indicator	Malawi	Ghana	Kenya
Non-commodity delivery cost/dose, financial / economic [32]	$1.04 / $1.52	$2.30 / $4.09	$2.46 / $4.62
Total cost/four-dose completion, financial / economic [32]	$10.86 / $35.16	$15.07 / $45.24	$21.30 / $51.49
Reported staff time/vaccination, fixed / outreach sessions [32]	6 / 6 min	9 / 9 min	14 / 12 min
Additional cold-chain requirements reported [32]	None	29 refrigerators; 180 cold boxes	83 refrigerators

Interpretation: Base-case estimates from Table 3 of Baral et al. [32]; staff time and infrastructure from Table 1 of that study. Delivery costs exclude vaccine and immunization supplies. Economic totals value donated vaccine at **$5/dose**; financial totals exclude donated vaccine acquisition but include procurement add-ons. **Wastage was assumed at 10%**, not measured. Staff time was survey-informed, not a staffing norm. Fourth-dose eligibility was incomplete during observation; these completion estimates are not mature-programme benchmarks. International donor technical-assistance costs were excluded [32]. Four-dose completion is a service endpoint, not guaranteed protection.

B. Product evidence, projections and policy

Product / setting	Evidence category	Verified statement	Permitted interpretation and limit
R21/Matrix-M; modeled sub-	**Model projection**	With an assumed $3 vaccine-dose price, reported incremental cost/DALY	Conditional modeled value; not measured country expenditure,

Saharan African settings		averted was $34 (range $29–139) in perennial settings and $30 ($22–172) in seasonal settings [88]	an affordability threshold or evidence of equitable access
R21/Matrix-M; Gavi–UNICEF announcement, November 2025	**Announced procurement term**	$2.99/dose, anticipated approximately one year later [89]	Prospective agreement; not an observed universal price or delivered-schedule cost
R21, single-dose presentation; Indian manufacturer, WHO product record	**Documented product specification / regulatory milestone**	Ready-to-use liquid; 2–8 °C; 24 months; 14.06 cm^3/dose in secondary packaging; prequalification effective 19 December 2023 [33]	Presentation-specific logistics and a dated milestone; not measured country cold-chain capacity or regulatory review duration
R21, two-dose presentation	**Documented handling requirement**	Opened vials discarded within six hours or at session end, whichever occurs first [34]	A potential determinant of wastage; not an observed wastage percentage
eOD-GT8 60mer clinical material	**Clinical manufacturing/stability evidence**	Clinical-grade manufacture and −80 °C storage reported [75]	Clinical supply example; not a commercial cost-of-goods estimate or evidence that standard refrigeration is adequate
mRNA technology-transfer investment report	**Stakeholder accounts and modeled scenarios**	Financing/workforce needs and production-capacity scenarios discussed [93]	Describes requirements and possible futures; does not establish candidate-specific costs or realized access gains
East African Community medicines initiative	**Documented regional experience, indirect to these vaccines**	Overview reports approximately halved average registration time in its pilot process [94]	Not a country-specific nanovaccine approval-time estimate

African vaccine manufacture	**Historical baseline and policy target**	Less than 1% locally manufactured at the framework's baseline; 60% target for 2040 [96]	Neither is a current candidate-specific supply or equity outcome
Within-country completion, household costs and access inequalities	**Proposed evaluation priorities**	Outcomes to measure for the intended product and population	No achieved equity benefit is asserted

Table-wide limitations: These rows answer different questions and should not be pooled into a single affordability or readiness score. No audited commercial manufacturing cost or comparable candidate-specific national regulatory duration is established by this table. Product storage requirements are not evidence that all delivery sites can meet them. Model ranges are not automatically confidence intervals. Dollar values from different years or cost boundaries are not directly comparable.

Table S3. Geographic, exposure and host characteristics of the disease-specific clinical evidence discussed

**Candidate / study**	**Recruitment region and endemicity / exposure context**	**Prior exposure, infection or serostatus**	**Age and relevant host characteristics**	**Evidence supported and extrapolation to Global South implementation**
**RTS,S/AS01, phase 3 [22]**	Sub-Saharan Africa: Burkina Faso, Gabon, Ghana, Kenya, Malawi, Mozambique and Tanzania; 11 sites with heterogeneous malaria transmission.	Naturally exposed endemic populations; not a malaria-naïve cohort. Individual exposure histories are not standardized across sites.	Separate cohorts aged **5–17 months** and **6–12 weeks** at enrolment. The Table 1 headline estimate refers to the older cohort with a booster.	Direct pediatric malaria efficacy evidence in sampled African settings. Does not establish the same effect in other ages, transmission settings or regions.
**R21/Matrix-M, phase 3 [23]**	Burkina Faso, Mali, Kenya and Tanzania; seasonal and perennial sites, with seasonal or age-based administration.	Endemic-exposure cohort, not selected as malaria-naïve; local exposure and background prevention vary.	Children aged **5–36 months**; efficacy in children with HIV was not assessed in this trial.	Direct efficacy evidence in selected African children. Transfer to other exposure patterns or host groups requires separate assessment.

**R21/Matrix-M, phase 1/2b follow-up [58]**	Nanoro, Burkina Faso; highly seasonal malaria setting.	Naturally exposed children; seasonal vaccination context.	Children aged **5–17 months** at initial enrolment.	Supports durability and booster findings in this cohort; does not establish equivalent durability across the Global South.
**RH5.1/Matrix-M, phase 2b [28]**	Nanoro, Burkina Faso; one seasonal-transmission setting.	Naturally exposed children; individual prior exposure is distinct from residence in an endemic area.	Children aged **5–17 months**.	Interim pediatric efficacy evidence in one setting; wider geographic or host-population applicability remains unestablished.
**RH5.1/Matrix-M, phase 1b [54]**	Bagamoyo, Tanzania; malaria-endemic setting.	Different malaria pre-exposure groups were studied, including children with higher pre-exposure.	Healthy adults aged **18–45 years** and children aged **5–17 months**.	Direct local safety/immunogenicity evidence. Functional antibody assays do not establish clinical efficacy in these groups.
**M72/AS01E, phase 2b efficacy [21]**	Kenya, South Africa and Zambia; TB-endemic settings.	**IGRA-positive** *M. tuberculosis* infection; no active TB; baseline sputum PCR-negative.	**HIV-negative adults aged 18–50 years**.	Direct prevention-of-disease evidence in infected adults. Application to uninfected people, children or people living with HIV is extrapolation.
**M72/AS01E-4, phase 2 [55]**	South Africa; settings with substantial TB and HIV burden.	TB preventive therapy completed; HIV status and treatment characterize this separate cohort.	Participants aged **16–35 years** with well-controlled HIV, receiving ART and with viral suppression.	Direct safety/immunogenicity evidence in selected people living with HIV; does not demonstrate TB disease-prevention efficacy or cover uncontrolled HIV.
**ID93/GLA-SE, phase 1 [42]**	United States; selected volunteer cohort rather than an endemic-	**BCG-naïve, QuantiFERON-negative**.	Healthy adults; 60 enrolled.	Human safety/immunogenicity and adjuvant-comparator evidence. Transfer to previously infected or

	population efficacy trial.			BCG-vaccinated endemic populations is extrapolation from this cited trial.
**H1/CAF01, phase 1 [48]**	Leiden, Netherlands; low-incidence TB setting.	BCG-naïve volunteers without evidence of TB infection.	Healthy adults aged **18–55 years**.	Early safety and sustained T-cell findings. Does not establish protection in endemic, previously infected or BCG-vaccinated populations.
**eOD-GT8 60mer/AS01B, IAVI G001 [29]**	United States; phase 1 volunteer study, not an HIV-acquisition efficacy trial.	HIV-uninfected.	Healthy adults aged **18–50 years**; 48 enrolled.	Direct US evidence of precursor activation. Extension to other populations or protection against HIV acquisition remains unproven.
**mRNA-encoded nanoparticles, IAVI G002 [30]**	United States; early-phase immune assessment.	HIV-uninfected.	Healthy adults; eligibility **18–50 years**; 60 enrolled.	US priming/boosting evidence. Does not establish protection or transferability of the complete regimen to African populations.
**mRNA-encoded nanoparticles, IAVI G003 [30]**	Rwanda and South Africa; substantial regional HIV burden, but no acquisition-efficacy assessment.	HIV-uninfected.	Healthy adults; eligibility **18–50 years**, enrolled ages **21–41**; 18 enrolled.	Direct African priming evidence, not efficacy or representativeness of all African populations. Does not reproduce the full G002 heterologous-boosting evaluation.
**PepGNP-Dengue, phase 1 [31]**	Lausanne, Switzerland; non-endemic study setting.	**Dengue-naïve**.	Healthy adults aged **18–45 years**; 26 enrolled.	Early safety/exploratory cellular evidence. Transfer to dengue-exposed children or adults in endemic countries remains untested.

Table notes. Age ranges are enrolment or eligibility ranges unless otherwise specified; G003's actual enrolled ages are shown separately. Population descriptors apply to the cited study, not every trial of the candidate. Endemicity describes the setting and should not substitute for individual infection or serostatus. "Direct" denotes evidence in the sampled population, not comprehensive regional representativeness. This table

describes the disease-specific clinical studies underpinning the review; animal studies, surveillance reports, conventional-vaccine guidance and registered studies without reported outcomes are not treated as additional efficacy cohorts. Background COVID-19 examples are contextual platform evidence, not protection evidence for the four target diseases.

Abbreviations: ART, antiretroviral therapy; BCG, Bacillus Calmette–Guérin; HIV, human immunodeficiency virus; IGRA, interferon-gamma release assay; PCR, polymerase chain reaction; TB, tuberculosis.

Table S4. Descriptive scope of literature identification, purposive source selection, and critical narrative synthesis.

Scientific-literature window: 1 January 2016–30 April 2026, with selected earlier foundational studies and separately identified contextual documents. The reported revision searches were performed on 5 September 2026. Keyword groups below are descriptive, not reconstructed complete Boolean strings. The search date and publication cutoff serve different purposes.

Source or activity	Reported scope	Reporting limits
Existing bibliography	Pre-existing disease-specific and contextual references, supplemented by targeted searches and source checks.	Complete historical strategies and selection-stage dates are not reported.
PubMed revision query 1	PubMed via NCBI E-utilities; title/abstract fields. Named clinical candidates + trial-related terms; publication-date window applied. Reported retrieval: 88 records.	Exact term list and full Boolean string are not reported. Count is query-level.
PubMed revision query 2	PubMed via NCBI E-utilities. mRNA + manufacturing or technology transfer + Global South or low- and middle-income terms; publication-date window applied. Reported retrieval: 16 records.	Full Boolean string and fields are not reported. Query overlap is unquantified.
Individual source verification	Publisher/Crossref, PubMed/Europe PMC and accessible texts; selected WHO, UNAIDS, Africa CDC, PAHO, Gavi–UNICEF and ClinicalTrials.gov documents.	Claim-specific checks, not systematic organizational searches. No systematic Scopus, Web of Science or registry search claimed.
Implementation and product checks	Targeted revision checks: RTS,S pilot costing, WHO R21 product record and R21 package insert [32–34].	Contextual checks do not extend the scientific-literature window or establish exhaustive coverage.
Purposive relevance assessment	Malaria, TB, HIV and dengue; functional nanoscale components. Priority: human clinical outcomes, component comparisons, in vivo findings and candidate-specific mechanisms; relevant implementation sources.	Purposive selection; no dual screening. Global South implementation focus, without restricting evidence origin.

Exclusions and contextual evidence	Non-vaccine nanotherapies, duplicates and insufficient-detail conference abstracts excluded from disease-specific evidence. In vitro-only findings without further translational evidence excluded as efficacy evidence.	Other-disease/tumor examples are labeled context. Historical exclusion counts are not reconstructed.
Critical appraisal and synthesis	Qualitative appraisal by design, model, population, comparator, endpoint and follow-up. Table 1: clinical evidence; S1: mechanisms; S2: implementation; S3: populations.	Evidence levels and observations/models/targets separated. No pooling, normalized efficacy scale, platform ranking or formal risk-of-bias score.

Notes. The totals of 88 and 16 are not unique screened or included-study counts. Historical screening and final inclusion totals are not reconstructed; the reference list is not an included-study count. Searches reportedly applied no language filter, but English-readable source reliance may introduce bias. This unregistered narrative review makes no PRISMA or meta-analysis claim; selection and publication bias remain possible.

Abbreviations: HIV, human immunodeficiency virus; mRNA, messenger RNA; NCBI, National Center for Biotechnology Information; PAHO, Pan American Health Organization; PRISMA, Preferred Reporting Items for Systematic Reviews and Meta-Analyses; TB, tuberculosis; UNAIDS, Joint United Nations Programme on HIV/AIDS; WHO, World Health Organization.
